# What do we believe in? Rumors and processing strategies during the COVID-19 outbreak in China

**DOI:** 10.1177/0963662520979459

**Published:** 2020-12-22

**Authors:** Wenxue Zou, Lu Tang

**Affiliations:** Texas A&M University, USA

**Keywords:** China, COVID-19, information processing, interview, rumor

## Abstract

The COVID-19 pandemic is called the first infodemic in history. Those first confronted by the enormous challenge of fighting this infodemic to save their lives were the people of Hubei Province in China. To understand how they defined and processed rumors, we conducted an interview study with Hubei residents when they were under lockdown. We found that they typically defined rumors in terms of one or two of three features: non-factual information, information unsanctioned by the government, and information causing panic. They reported low motivation in verifying the information and often either rejected any information they perceived as suspicious or waited for the government to debunk rumors. Even among those who tried to verify information, most relied exclusively on heuristic processing cues such as source credibility, linguistic and visual cues, and intuition. Systematic processing strategies such as fact-checking and discussing with family and friends were seldom used.

On the evening of 31 January, 2020, one week into the lockdown of Wuhan due to the COVID-19 outbreak, news media and social media in China simultaneously shared one news story: a joint research study by the Shanghai Institute of Materia Medica and Wuhan Institute of Virology found that double coptis (*shuang huanglian*), a traditional Chinese herbal medicine commonly used to treat cold and flu, can effectively contain the multiplication of SARS-CoV-2, the virus causing the COVID-19, in human bodies. Within hours, people swarmed pharmacies, and this medicine was completely sold out throughout China overnight. However, within a few days, the national ecstasy was dampened by the news that this medicine was not the magical cure. Considered one of the largest debacles in China’s efforts to battle the COVID-19 outbreak, the double coptis incident was directly caused by the spread of misleading information. However, it was only one of the rumors (*yaoyan*) or misinformation (*bushixinxi*) circulated during the early stage of the outbreak in China.^1^

Rumors are a natural by-product of any crisis. The ongoing COVID-19 pandemic is different from previous outbreaks of emerging infectious diseases such as SARS, MERS, or Ebola because it is characterized by an avalanche of rumors, misinformation, and conspiracy theories. As a result, the COVID-19 pandemic has been called the first infodemic the world has witnessed (Zarocostas, 2020).

This study examined how residents of Hubei Province, the epicenter of the COVID-19 outbreak in China, described and defined rumors through in-depth interviews conducted when they were under lockdown in early 2020. Special attention was paid to the heuristic and systematic information processing strategies used to decide if a piece of information is a rumor. Theoretically, the study dissected the highly politicized definition of rumor in China during a public health crisis as a form of discursive control. Practically, our findings can guide the creation of effective risk communication in China’s unique context.

## 1. Literature review

### Rumor and misinformation about science and medicine

Numerous definitions of rumor have been proposed. Some researchers define rumors as information that is not verified or not based on facts (e.g. Allport and Postman, 1947; Knapp, 1944; Sunstein, 2014). Knapp (1944) identified three types of rumors: pipe dreams (wishes and desires), bogies (fears), and wedge drivers (rumors dividing people and undermining relationships). Others consider rumor a form of collective problem-solving at a time of uncertainty (Fisher, 1998). Rumors fulfill specific cognitive needs by allowing the public to make sense of an ambiguous situation together and help people cope with emotions such as fear, anxiety, and uncertainty (Rosnow, 1988).

Recently, the terms misinformation and disinformation are more commonly used to describe non-factual information, especially communicated on the Internet and social media. Misinformation usually focuses on a message’s truthfulness. Disinformation is used to describe the sender’s malicious intention to deceive, manipulate, and defeat (Fetzer, 2004).

Researchers have started to examine the rumors and misinformation about science and medicine, such as genetically modified foods (GMOs), nuclear energy, vaccines, and climate change. Many studies examined the contents of such rumors and misinformation. For instance, Sell et al. (2020) examined tweets during the Ebola 2014 outbreak and found that 10% of the tweets contained false or partially false information. Rumors about government conspiracies, promoting public discord, and elevating fear are most common. Sommariva et al. (2018) analyzed social media content about the Zika virus and identified two major types of misinformation: conspiracy theories and information downplaying the virus’ risks.

Some researchers have explored the relationship between individuals’ psychological characteristics and their emotional and behavioral responses to rumors and misinformation about science and medicine. Overall, epistemologically naive users are more likely to share online rumors than epistemologically robust users (Chua and Banerjee, 2017; Li and Sakamoto, 2015). Subjective knowledge, that is, the belief in one’s knowledge about a scientific subject, also influences one’s response to scientific misinformation. Kirkpatrick (2021) found that while perceived risk predicted the audience’s likelihood of sharing scientific misinformation, this effect was stronger among those with higher subjective knowledge. Emotion also plays a role in people’s response to health rumors. Na et al. (2018) found that those who already felt angry were more likely to believe anger-inducing rumors during a health crisis.

Recently, researchers have started exploring the macro-level dissemination patterns of rumor and misinformation about science and medicine on social media. For instance, Xu et al. (2020) studied the spread of misinformation about GMOs on Sina Weibo through social network analysis and found that the dissemination network of misinformation is denser and thus structurally more stable than the network of accurate information. This finding means misinformation gets spread more efficiently than scientifically accurate information. Also using social network analysis, Tang et al. (2020) studied the exposure to anti-vaccine misinformation on YouTube based on the platform’s recommendation algorithms. This study found that viewers were more likely to encounter anti-vaccine videos when they start with an anti-vaccine seed video than starting with anti-vaccine search keywords, and misinformation begot more misinformation.

However, little is known about the strategies people used in processing science and medicine-related misinformation and rumors. The heuristic–systematic model (HSM) is a useful theoretical framework for this purpose.

### The heuristic–systematic model

The HSM was initially developed to explain individuals’ behavior changes based on two modes of information processing: systematic processing and heuristic processing. Systematic processing refers to active attempts to understand and evaluate the arguments in a message, including keen observations, in-depth deliberation, and careful reasoning, while heuristic processing requires less cognitive efforts and relies on easily accessible cues such as source credibility and communicators’ group membership (Chaiken et al., 1989). The least effort principle and the sufficiency principle were used to explain the choice of processing modes (Chaiken et al., 1989). Unlike heuristic processing, systematic processing makes high demands of individuals’ critical thinking capability, perceived information efficacy, and the motivation to apply cognitive resources (Eagly and Chaiken, 1993; Griffin et al., 2002). The least effort principle states that individuals prefer the mode requiring the least effort (i.e. heuristic processing). Simultaneously, the decision is also based on the sufficiency principle, which means individuals need to achieve a balance between the least efforts and sufficient confidence. The HSM identifies three types of motivation: accuracy motivation (the need to process the information accurately), defense motivation (the desire to form judgment consistent with one’s beliefs and interests), and impression motivation (the need to form judgment consistent with one’s social goals). The HSM has been used to study people’s attitudes toward health risk information. Research indicates that one’s information sufficiency gap, knowledge structures and level of issue involvement, information characteristics, and communicators’ credentials influence their processing mode choices (Griffin et al., 2002; Kahlor et al., 2003). The HSM can also guide the study of how people process COVID-19-related information.

### Rumor, censorship, and public opinion control in China

In the Chinese language, the word “*yao yan*” is the equivalent of rumor. The word *yao* was found in *The Book of Songs*, the country’s oldest collection of poems to refer to folk songs. Gradually, the word *yao* was used to describe gossips unsupported by fact. Instead of treating *yao* as something evil or dangerous, ancient rulers considered *yao* an expression of public opinion and had officials collect them and report to the emperors (for a more detailed discussion, see Tao, 2014). In *Origins of Words* (*Ci Yuan*), an authoritative dictionary published in the early twentieth century, the word “*yao yan*” was defined as either political folk songs circulated among the public or gossip unsupported by fact. However, in the *Collection of Words* (*Ci Hai*), another authoritative dictionary amended in the 1960s by the current Chinese government, the definition of political folk songs was replaced with folk songs, and political connotation was taken out (Deng, 2020).

Promoting the slogan of “Don’t create, believe or spread rumors” (*bu zaoyao, bu xinyao, bu chuanyao*), the Chinese government has issued a series of laws and regulations in recent years to specify the standards of punishment. In 2013, China’s Supreme Court and Prosecutor General’s Office issued a new explanation of the country’s Criminal Code, dictating that spreading defaming rumors online would be considered libel and a serious criminal offense if the original message was viewed more than 5000 times or reposted for more than 500 times (Blanchard et al., 2013). In 2015, China amended its Criminal Code and added the following item: creating false information about disasters, disease outbreaks, and policing and spreading such information on the Internet and other media or doing this knowingly to disturb social order will be punished with 3-year imprisonment or less. Particularly serious offenses can be penalized with 3- to 7-year sentences (Human Rights Watch, 2015).

During the COVID-19 outbreak, the Chinese government has been particularly focused on the control of rumors. During the early days of the outbreak, news about this unknown virus was suppressed, and human-to-human transmission was denied. The government officially acknowledged human-to-human transmission on 20 January and put Wuhan under lockdown on 23 January. On 25 January, WeChat, China’s dominant social media platform boasting over 1 billion monthly users, published a statement to reinforce the Criminal Code about online rumors described above. It announced that it would “delete unlawful information and escalate the punishment of account holders based on the severity of the violation, including but not limited to temporary and permanent suspension” (Sun, 2020). On 6 February 2020, the Chinese government announced that spreading rumors about the outbreak can be punished as libel (China’s Health Commission, 2020). It is within this context that we ask the following research questions (RQs):

*RQ1.* What were the major rumors residents of Hubei Province encountered during the COVID-19 outbreak?
*RQ2.* How did they define rumors related to the COVID-19 outbreak?
*RQ3.* What strategies did they use to ascertain the quality of information about COVID-19?

## 2. Method

### Participant recruitment

Convenient sampling was used for participant recruitment. After obtaining Institutional Review Board approval, the first author, who grew up in Hubei Province, reached out to her acquaintances to identify potential participants. Snowball sampling was used to recruit additional participants. We tried to achieve a balance in terms of gender, age, and place of residence in Hubei Province. In the end, 17 participants (8 men and 9 women) were recruited. These participants ranged from 26 to 54 years in age. Five participants were living in Wuhan, the capital city of Hubei Province, and 12 were from 6 other cities in the province. Each participant was given a pseudonym, and their demographic information is presented in Table 1. None of them contracted COVID-19 at the time of data collection. Participants were not compensated.

**Table 1.** Demographic information of participants.

No	Alias	Age	Sex	Education	Occupation	Residence
1	Ling	26	F	Master’s	Financial advisor	Huangshi
2	Hui	56	F	High school	Retired	Daye
3	Cheng	26	F	Bachelor’s	Pharmaceutical sales	Huangshi
4	Guo	50	M	Bachelor’s	IT staff at a bank	Huangshi
5	Mei	27	F	Master’s	Game developer	Huangshi
6	Ming	38	F	Some college	Barbershop owner	Jinniu
7	Hong	26	F	Master’s	New media marketing	Xiangyang
8	Feng	51	M	High school	Construction company supervisor	Wuhan
9	Ding	27	M	Master’s	Bank staff	Wuhan
10	Liang	24	M	Bachelor’s	Graduate student	Wuhan
11	Guang	54	M	High school	Project manager at an engineering company	Wuhan
12	Lili	35	F	Bachelor’s	Financial advisor	Wuhan
13	Jin	51	F	High school	Owner of a truck tire maintenance shop	Wuhan
14	Jay	24	M	Bachelor’s	Mechanical engineer	Xianning
15	Wu	26	F	Master’s	New media marketing	Xiangyang
16	Huang	27	F	Master’s	Human resource staff	Huangshi
17	Lei	27	M	Master’s	Information system designer	Huanggang

### Data collection

Semi-structured interviews were used to ask flexible and exploratory, open-ended questions to obtain participants’ perceptions of rumors and strategies used to distinguish the truth. The first author conducted all the interviews using the voice chat function of WeChat. Initially, we asked participants how they defined rumor, but many participants could not offer a definition. So we asked them if they could recall rumors they encountered during the COVID-19 outbreak. Then we asked them to reflect on these examples and explain why they were rumors. Finally, we asked participants to describe the methods they used to evaluate a piece of information to decide if it was a rumor. This study was a part of a larger study examining information and media use in China during the COVID-19 outbreak. Results based on additional questions were reported elsewhere (Tang & Zou, 2020). Interviews typically lasted between 35 and 50 minutes. All interviews were recorded with the permission of participants and transcribed verbatim by the first author. All interviews were conducted in Mandarin Chinese.

### Data analysis

**Figure 1.**
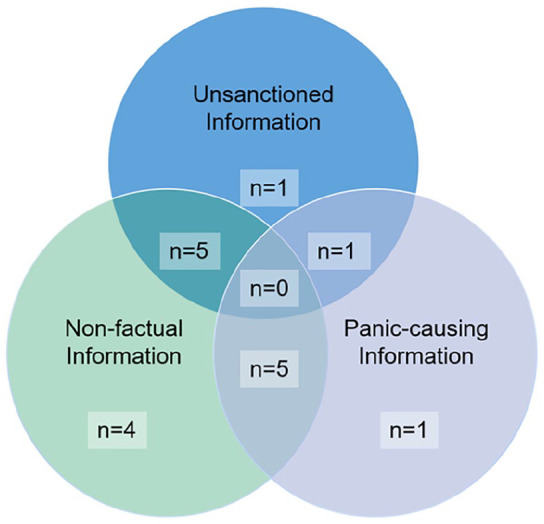
Definitions of rumors.

**Figure 2.**
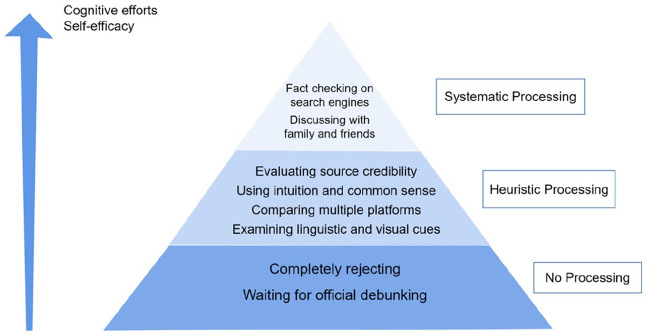
Strategies to identify rumors.

We used the phronetic iterative approach in data analysis. The phronetic iterative approach is an interpretive method for data analysis that prioritizes the understanding of contextual knowledge (Tracy, 2018). First, we conducted open coding similar to the first step of grounded theory building and identified descriptive codes from the transcripts (Glaser and Strauss, 1967). To answer RQ1 (types of rumors), we used the constant comparison method and collapsed the rumors identified by participants into major categories. To explore RQ2 (definition rumor) and RQ3 (information processing strategies), we conducted a secondary cycle analysis to create a more in-depth understanding of the transcripts. We paid particular attention to the characteristics of rumors based on existing theories about rumor communication and the strategies of information processing based on the HSM. For RQ2, we identified three defining characteristics of rumor (non-factual information, unsanctioned information, and panic-causing information). We then applied this typology to content analyze the transcripts again, which allowed us to tabulate the frequencies by which different dimensions of rumor were mentioned by participants to understand further the relationships among these three defining characteristics (see Figure 1). To answer RQ3, we categorized participants’ responses into three categories: no processing, heuristic processing, and systematic processing (see Figure 2). The data analysis was conducted in Chinese to preserve the interviews’ culturally specific terms and subtle cultural assumptions. Selected quotes were translated into English for this article.

### Validity

Several measures were taken to ensure the quality of our research. Our study’s credibility was established through prolonged engagement and persistent observation (Lincoln and Guba, 1985). Both authors grew up in China, and even though we currently live abroad, we were regular users of Chinese social media apps such as WeChat and Weibo. During the COVID-19 outbreak in China, we spent an extensive amount of time browsing Chinese social media and websites to stay updated with the outbreak and observe how people in China use different media to share information about the outbreak and express their opinions and emotions. We encountered some of the rumors ourselves during this process. To ensure the validity of the interpretation of the stories, meanings, and symbolism, we also used peer debriefing by inviting several scholars specializing in Chinese culture and communication to critique an earlier draft of the article (Marshall and Rossman, 2010).

## 3. Results

### RQ1: Major types of rumors encountered

Participants all recalled rumors they encountered during the outbreak. These rumors can be grouped into the following categories: (1) rumors about the number of confirmed cases of COVID-19 and the situation at the frontline, (2) rumors about how the virus can be transmitted, (3) rumors about methods of prevention and treatment, and (4) rumors about the government’s (mis)handling of the crisis.

#### Rumors about the number of confirmed cases and the situation at the frontline

Many participants reported rumors about the number of confirmed cases. Because of the difficulty in precisely reporting the number of cases and the government’s information control, very little information about the scale and severity of the outbreak was available to the public initially. As a result, rumors about the numbers of confirmed cases and death were widely circulated on social media. For example, Ling (P1, 26-year-old woman) mentioned, “There was a rumor that all 120 residents of the Wuhan West Road Nursing Home have contracted COVID-19. [In fact], as of yesterday, only eight people were infected, and all of them are being treated [in hospitals].” Wu (P15, 26-year-old woman) and Lei (P17, 27-year-old man) both recalled that they saw pictures and videos taken by citizen journalists in hospitals and funeral homes showing large numbers of dying or deceased patients or videos of people dropping dead on the streets of Wuhan.

#### Rumors about transmission routes

Because of the novelty of the SARS-CoV-2, enormous uncertainty existed in terms of how it was transmitted. Many participants believed that they were exposed to rumors about how the virus was transmitted and how long the incubation period was. Initially, the official party line was that the virus could only be transmitted from animals to humans. Even after the Chinese government acknowledged human-to-human transmission on 20 January, the public was still unsure about the routes of transmission. Escalating news that it could be transmitted through contact, droplets, aerosols, or fecal matters continued to surface. For instance, Mei (P5, 27-year-old woman) reported seeing the news that the virus can be spread through aerosol transmission and considered it a rumor. Hong (P7, 26-year-old woman) recalled the rumor that the virus could survive in drinking water. Mei (P5) also recalled a rumor that the incubation period could be as long as 30 days.

#### Rumors about prevention and treatment methods

Rumors about methods of prevention and treatment were abundant throughout the outbreak. In addition to the rumor about the effectiveness of double coptis discussed earlier, several participants recalled the news about the successful development of a COVID-19 vaccine. On 25 February, every news media and social media reported that a research team at Tianjin University had successfully developed a vaccine based on food-grade yeast, which could be taken orally. The lead researcher had tested the vaccine on himself and found it to be safe. Even though this news looked suspicious for people with any basic knowledge about medicine and vaccines, the said vaccine was hailed as a miracle cure until the news was debunked on the following day. Other participants mentioned even more absurd rumors about prevention and treatment. For instance, Cheng (P3, 26-year-old woman) said, “there was a rumor circulating on Tencent News (a major online news portal in China) that eating 12 boiled eggs in one sitting can prevent COVID-19.” Some participants rejected any claim of quick cures. For example, Guang (P11, 54-year-old man) concluded, “Any claim of a quick cure [of COVID-19] is definitely a rumor.”

#### Rumors about the government’s (mis)handling of the outbreak

The last major category of rumors was about the ways the provincial and local governments handled or mishandled the outbreak. Ling (P1, 26-year-old woman) provided two examples. First, it was said that auxiliary police officers confiscated the vegetables donated to the residents of Wuhan by the farmers in Sichuan Province. She went on to say, “the truth was that they were simply helping unload the vegetables, but people believed in this rumor because they always hated the auxiliary police.” Another piece of rumor identified by Ling was the news that the High Court of Shanghai arrested residents for buying too many infrared forehead thermometers from pharmacies. She said, “this is completely fabricated to create a negative feeling among people.”

Several participants spoke of Dr Li Wenliang, an ophthalmologist at Wuhan Central Hospital. Dr Li warned his co-workers of a highly dangerous SARS-like virus in a WeChat group on 30 December 2019. He and seven other doctors who also shared similar information were reprimanded by the local police department for spreading rumors and creating panic. They were considered whistleblowers and were lovingly called “The Virtuous Eight” (*ba junzi*) by the people. Dr Li later contracted COVID-19 and passed away. Information regarding Dr Li was highly censored on China’s social media.

### RQ2: Definitions of rumor

Based on the examples of rumors provided by our participants and how they analyzed them, we found three prominent characteristics participants used to define rumors: non-factual, unsanctioned, and panic-causing. Sometimes, individuals defined rumors using more than one of these features. Age was often related to the definition adopted. (See Figure 1 for the frequencies by which our participants mentioned each concept.)

#### Non-factual information

Most young participants regarded rumors as non-factual information. For instance, Hong (P7, 26-year-old woman) said, “Rumors are non-factual hearsay.” Similarly, Liang (P10, 24-year-old man) said, “whether a message is a rumor is based on whether it tallies with the facts. For example, some people say that [this new coronavirus] is SARS, but is it exactly the same as SARS?” According to this definition, rumors were equivalent to misinformation in the eyes of the young participants.

#### Information unsanctioned by the government

Some participants (including participants of all ages) considered any information unsanctioned by the government to be rumors. For instance, when asked how she evaluated the warning sent out by Dr Li and other whistleblowers early on, Jin (P13, 51-year-old woman) said,

at that time, I thought it was a rumor because the government said the virus would not spread from person to person. Many people said the police arrested some doctors. So I thought [the warning] was a rumor and didn’t dare to pass it on.

Jin went on to say, “I heard that if people spread this rumor, their WeChat groups will be disbanded, their WeChat accounts will be suspended, and the money they saved on WeChat Pay would be lost. So I did not dare to say anything.” At the time of data collection, what Dr Li said was proved to be accurate; however, some participants still believed Dr Li spread a rumor because he should not have shared this information outside of the official channel. For instance, Guo (P4, 50-year-old man) said,

I think it belonged to rumors. [. . .] If you were a doctor and you noticed a new virus, you should report it through proper channels, instead of telling the public, who did not have the expertise to evaluate the information. They wouldn’t understand what you were talking about.

#### Information leading to panic

Different from younger participants, many older participants defined rumor in terms of its negative effects or senders’ evil intentions. Some defined rumor in terms of its effect on the public and labeled any information that creates panic rumor. For instance, Hui (P2, 56-year-old woman) held, “I consider any information that makes me feel hopeless and disappointed as unlikely to be true; so I treat them as rumors.” Echoing her sentiment, Jin (P13, 51-year-old woman) identified rumors as “what people use to scare others.” Some participants defined rumor in terms of the senders’ intention. Ming (P6, 38-year-old woman) called people spreading rumors “detractors.” She pointed out that “detractors created rumors to harm others’ interests or crush their hopes.”

As shown in Figure 1, while some participants had a one-dimensional definition of rumor (non-factual = 4, unsanctioned = 1, and panic-causing = 1), most participants defined rumor using two of the three criteria. Some participants (n = 5) believed that rumors were non-factual information that was unsanctioned by the government. The problem of this definition was information unsanctioned by the government was not necessarily false. To some, governmental approval was given priority. For instance, Jin (P13, 51-year-old woman) believed, “If the government declares a certain kind of medicine as effective, I will believe it. [. . .] But I do not believe in any COVID-19 medicine peddled on the Internet. They are all overblown, just like rumors.” To other participants, if a piece of information turned out to be factual, it was not a rumor, even though it did not have governmental approval. For instance, when asked whether Dr Li was spreading rumor when he first warned others of the risk of COVID-19, some participants were confident that he was telling the truth, even though he was punished for it. For instance, Liang (P10, 24-year-old man) said, “I don’t think it is a rumor.”

Besides, another group of participants (n = 5) defined rumor as non-factual information that causes panic. This group of participants was all in their twenties. Because they considered rumors as panic-causing, they were more motivated to process the information they had and use different strategies to verify potential rumors.

### RQ3: Strategies to identify rumors

Our participants identified several common strategies to determine if a piece of information is a rumor. We categorized these strategies on three levels based on the amount of cognitive efforts involved: (1) systematic processing, (2) heuristic processing, and (3) no processing (see Figure 2).

#### Systematic processing

Systematic processing occurred when individuals carefully considered the information they received and were motivated to gather additional information to evaluate the news. Systematic processing usually required substantial cognitive efforts (Yang et al., 2010, 2019).

##### Fact-checking through search engines

When unsure about a message, some participants would confirm the authenticity of the information using search engines. For instance, Mei (P5, 27-year-old woman) recalled, “If I am unsure about a piece of news [I encountered online], and the news story used some evidence, I would verify the evidence using search engines.” Similarly, Jay (P14, 24-year-old man) said, “I would first evaluate the message’s internal logic. If I still couldn’t decide afterward, I would go to Baidu [to search it].” Baidu is the most used search engine; however, an increasingly long list of sensitive keywords was blocked on Baidu and other search engines in China. As a result, participants (especially older participants) reported giving up their effort at fact-checking if they failed to find anything on Baidu. For instance, Hui (P2, 56-year-old woman) explained, “I also tried to find [the facts that I want to know] in Baidu and other news, but I couldn’t find it. [So I gave up].”

##### Discussing with family and friends

When feeling unsure about the information they received, some participants chose to discuss it with their family and friends. Hong (P7, 26-year-old woman) mentioned, “One of my ways of evaluating a message is to share it with others and see if they can help me verify it.” Similarly, Ming (P6, 38-year-old woman) also reported that she would consider how her relatives evaluated a message in family group chats. Discussing such information with one’s family and friends privately face-to-face or through private chat groups in WeChat was more common than discussing messages in public space on social media.

#### Heuristic processing

The above-mentioned strategies of systematic processing were seldom used. Instead, most participants relied on strategies of heuristic processing in evaluating the information they received. Heuristic processing occurred when individuals made a quick decision about a piece of information based on heuristic and superficial cues such as source credibility or the language used. Heuristic processing usually required minimal cognitive efforts (Kahlor et al., 2003; Yang et al., 2010).

##### Using intuition and common sense

Many participants relied on intuition or common sense to evaluate the information they received instead of using specific strategies that required time and effort. Using common sense meant comparing the new information with one’s experience and knowledge. Lili (P12, 35-year-old woman) said, “During this period, we can’t go out. [Therefore] whether messages were true or false didn’t matter to me. [. . .] I will judge the information credibility based on the knowledge I have and common sense.” Feng (P8, 51-year-old man under quarantine in Wuhan) further said he used his “cognitive abilities.”

##### Evaluating the sources of information

Source credibility was the most used heuristic cue in assessing the information about COVID-19. Participants routinely evaluated the information they received by examining the sources of such information. Two types of sources were examined: the person or organization who created the message and the person who passed on the message on social media. Almost all participants expressed their trust in official news authorized by the government. For instance, in reflecting on her initial response to the news about double coptis as a magical cure, Mei (P5, a 27-year-old woman) said, “I first heard of the news from *People’s Daily’s* online version. I immediately believed it. I didn’t question it at all.” She went on to describe how she processed this information:

I trusted the news from official governmental media implicitly. I even shared this news in my family’s WeChat group and told them to buy [double coptis]. But I only read the headline and didn’t even read the actual content because it was 11 pm [and too late].

In addition, medical experts representing the government also enjoyed high credibility. For instance, Guo (P4, 50-year-old man) said, “I believe what Dr. Zhong Nanshan said because he led China’s battle against SARS. Now he is more than 80 years old and has been to the frontline of Wuhan in person.” However, not all doctors were trusted. Cheng (P3, 26-year-old woman) said she would only trust Dr Zhong Nanshan, who represented the “official source,” but not other physicians who posted about COVID-19. She said, “the information shared by famous health influencers online (doctors) contained their personal views. So I do not completely believe in them, but only use what they say as reference.”

Participants also evaluated the information they received based on who reposted it. Participants tended to believe the messages if the person who reposted the original messages was considered reliable and knowledgeable. Many participants regarded WeChat “Moments” (*pengyou quan*) as a relatively reliable way to obtain the epidemic-related information because news on Moments had already been filtered by their contacts and friends. Mei (P5, a 27-year-old woman who recently finished graduate school) said,

My contacts on WeChat are mostly my professors and classmates. I believe that they are capable of filtering and evaluating information because of their training. So if they approve of a video, I will become more convinced that this video is true.

In addition, Liang (P10, 24-year-old man) added that “if [the reposter] is someone that I am very familiar with, I would believe the messages forwarded by him/her.”

##### Comparing information from multiple sources and platforms

After receiving similar information from multiple platforms, some participants would compare if the core information was communicated in the same way in these different outlets. For instance, Hui (P2, 56-year-old woman) said, “If multiple sources shared the same news, the stories were consistent and the statistics were comparable, I would consider the news to be true.” However, comparing information from multiple sources and platforms was not frequently practiced.

##### Examining the linguistic and visual features of a message

Some participants would examine the linguistic and visual features of the message. They believed that rumors were characterized by sensational languages. For instance, Liang (P10, 24-year-old man) said, “credible information should be carefully worded. For example, it should objectively describe a situation without creating panic, and it should not use a bunch of exclamation marks.” He went on to say, “My grandmother often reposted WeChat messages with lots of exclamation marks and very strong language. [I wouldn’t trust such messages].” Both Liang (P10, 24-year-old man) and Wu (P15, 26-year-old woman) evaluated the information they received by “judging if there were subjective elements between the lines.” Specifically, Liang explained, “If its wording is very restrained, and it quotes many professional medical studies and data come from journal articles, I will trust this message.”

Some participants used other technical or visual cues in assessing whether a piece of news was a rumor. For instance, many pictures and videos allegedly taken by anonymous citizen journalists about the outbreak were widely circulated online, and they were difficult to authenticate. In this case, participants focused on the visual cues in those pictures and videos. For example, Hui (P2, 56-year-old woman) said,

sometimes it was difficult to tell when the videos were made [and] whether it was taken during the COVID-19 outbreak. I would pay attention to whether people in these videos were wearing face masks. If they were not, then the video was not taken recently.

In addition, when assessing pictures, some young participants reported that they would check if these pictures had been digitally altered. The photoshopped images were usually regarded as rumors.

#### No processing

Some participants took a completely passive attitude toward the information they received. They avoided any cognitive efforts in evaluating the information by delaying processing the information and waiting for the government to either confirm or debunk the story. Alternatively, some would altogether reject a message based merely on emotion.

##### Relying on official debunking of rumors

Sometimes when people encountered a message, they would neither accept nor reject. Nor would they think about it. Instead they would simply wait for governmental officials or state media to confirm or debunk the information. This is an extremely passive way of evaluating the news. For example, Ming (a 38-year-old woman) showed a lack of efficacy in evaluating the information she received, saying,

when a piece of sensitive information comes out, we cannot decide if it is a rumor or not. We just wait for the government to debunk it. We will wait to see if the government labels it as a rumor. [. . .] Today, it is really difficult to decide if a piece of news is true. We cannot evaluate it.

##### Complete rejection

Occasionally, an individual would completely reject a piece of information if he suspected it fit the definition of a rumor without verifying it. For instance, as described above, some participants completely dismissed any claims of a quick cure (e.g. Guang, P11, 54-year-old man), and some participants completely rejected any panic-causing information (e.g. Hui, P2, 56-year-old woman).

## 4. Discussion

### Rumor as a form of control

A large variety of rumors were widely circulated during the COVID-19 outbreak in China, including pipe dreams (e.g. rumors about miracle cures and vaccines), bogies (e.g. rumors about elevated risks, number of confirmed cases, and number of death), and wedge drivers (e.g. rumors about government’s mishandling of the outbreak). Our analysis shows that the concept of rumor, at least in the context of the COVID-19 outbreak, is constructed within China’s specific social, cultural, political, and legal contexts. While some participants define rumor in terms of the truthfulness of the information, others define rumor in terms of source (whether the government sanctions it), the sender’s malicious intention, and the resulting panic among receivers.

Our findings suggest that rumors about health and scientific medical knowledge can be highly politicized. When people define rumor in terms of whether the information is approved by the government, they surrender the power of defining what is true and what is false to the state. This surrender is the result of an ideology created by the state through legal and cultural apparatus in the last few decades. As a result, the state can reject any information that causes them inconvenience as a rumor and punish the source of such information (e.g. the case of Dr Li Wenliang). Our data show that the public is highly aware of the legal ramifications of spreading information from non-governmental sources and actively engage in self-censorship. This system of control resembles Michel Foucault’s concept of panopticon, in which people feel that they are always being watched (Foucault, 1995).

Similarly, defining rumor in terms of the sender’s malicious intent or the resulting panic is also highly problematic. In the context of the COVID-19 outbreak in China, pieces of information about the severity of the crisis are sometimes considered destabilizing rumors. Spreading such information is not only illegal but also immoral. Both our participants and social media in China used the idiom “eating steamed buns soaked in human blood (*chi renxue mantou*).” This term was initially coined by Lu Xun, an influential writer in the early twentieth century, to criticize the uncivilized and unsympathetic peasants who believed that eating steamed buns soaked with human blood could cure tuberculosis. However, in recent years, this idiom was appropriated to criticize those people who profit from others’ misery, especially by publicizing other people’s tragedies online. During the COVID-19 outbreak, those people who post sad human interest stories, such as how some patients were very ill but could not get treatment, were criticized for “eating steamed buns soaked in human blood.” For instance, Liang (P10, 24-year-old man) used this term to refer to people who “create panic by telling [the public] that a lot of people died and corpses cannot be transported fast enough.” Fang Fang is a novelist and resident of Wuhan who wrote the famous *Wuhan Diary* about her experiences of Wuhan lockdown and the sufferings of its people; but she was criticized for “eating steamed buns soaked in human blood” because she allegedly gained fame from other people’s sufferings by writing blogs and a book and handed ammunition to hostile foreign powers.^2^ Defining rumor as panic-causing information can silence the pain and suffering of individuals and, as a result, prevent any criticism of the government’s mishandling of the outbreak. Besides, suppressing or rejecting fear-causing information about the severity of the illness and the scale of the outbreak also contributes to the lack of timely adoption of prevention methods by the public, especially during the early stage of the outbreak.

Finally, many participants define rumor as information that is not supported by evidence or information that is not factual. This is similar to the notion of misinformation. Those who believe in this definition of rumor are more likely to use different strategies to verify the information they receive.

### Differentiating truth from rumors

Participants show very limited motivation and capability in differentiating truth from rumors, even though they are more educated than average Chinese citizens. Cheng and Lee (2019) label the current lack of interest in objective facts in China “the post-truth era.” Many participants do not feel motivated to critically analyze the information they receive, even during an unprecedented and scary public health crisis. Those who define rumor as information unsanctioned by the government do not feel the need to verify the truthfulness of the information. Instead, they prefer to wait for the government to tell them whether the information is true or not. Some participants define rumor as any information that causes panic and fear. In this case, they often completely reject any information that causes fear without trying to evaluate the content of the information. This can be dangerous during a public health crisis such as the COVID-19 outbreak when some useful information will inevitably be scary.

Among those who try to evaluate the information they receive, the majority only rely on heuristic cues as a cognitive shortcut, such as evaluating source credibility, examining linguistic and visual cues, comparing multiple platforms, and using intuition. Using these heuristic cues allows people to make quick decisions, but the results are not always accurate. For instance, some participants express that they absolutely trust the information coming from central and local governments and medical experts representing the governments. However, information from these sources is sometimes problematic.

Occasionally, some participants use systematic processing by fact-checking a message using search engines or discussing their information with others to evaluate the information collectively. However, the execution of these strategies is often hindered. As Baidu is the most popular search engine in China, people almost always use Baidu when they need to fact-check a message. However, like any other website and social media in China, Baidu has an ever-growing list of blacklisted keywords that cannot be searched. As a result, fact-checking on Baidu can often be futile, as indicated by a few participants. Discussing with family and friends represents a unique collective information processing method, whereby participants share the questionable information with those people they trust and hope to reach a conclusion together. This is probably an example of how China’s collectivist culture influences people’s processing of rumors. Such discussion is frequently mentioned as a way to make sense of COVID-19-related information when participants are under lockdown in Hubei Province. However, some individuals express fear in discussing sensitive information with their friends via WeChat, which is highly monitored and censored. Sending messages containing keywords the government considers to be unfavorable can lead to the suspension of WeChat accounts. Consequently, such discussion is often limited to private chats on social media or private face-to-face conversations.

Our findings tentatively suggest that older individuals are more likely to define rumor in terms of whether it causes public panic, and younger participants tend to focus on the information’s truthfulness. It is possible that older individuals have lower digital media literacy and are less capable of discerning the truthfulness of the information. As a result, they tend to focus on the consequence of rumor.

### Practical implications

Several practical implications can be drawn. First, since the government and governmental media are the primary sources people use in evaluating their information during the COVID-19 outbreak, they should scrupulously check and cross-check the accuracy of every piece of news when communicating health risk information. Governmental announcements with technical jargon should include explanations to promote comprehension. Second, since some people, especially older individuals, are likely to reject panic-causing information whether it is true or not, health agencies should strive for a balance between the fear appraisal and efficacy appraisal, as suggested by the Extended Parallel Process Model (Witte and Allen, 2000). Third, since many participants in our study reported a low level of efficacy in information processing, interventions to increase the public’s health literacy and media literacy are much needed.

### Limitations and directions for future research

One limitation of our study is that our participants recruited through convenience sampling are generally young or middle-aged, and most of them are college-educated. Furthermore, this study has a small sample size. Having a small sample size (usually less than 20) can facilitate the interviewers’ in-depth and fine-grained inquiries with the participants (Crouch and McKenzie, 2006). However, the small sample size prevents us from further comparing rumor perceptions among different age groups. Future studies could include a larger sample size or employ other methods such as surveys to further examine the relationship between age and how people define and process rumors. Furthermore, this study only investigated people’s understanding of rumors early on the outbreak; more research should be conducted to explore the public’s information processing mechanisms as the outbreak evolves.

## Notes

1. The terms of rumor and misinformation are often used interchangeably in China. Therefore, in line with common parlance we use “rumor” to refer to both.

2. Fang Fang’s case was not mentioned by any of our participants because it happened after our data collection.
